# 

**Published:** 1888-03-31

**Authors:** 


PRICE ONE PENNY.

[illegible]79, Vol. III.—March 31st, 1888.

[Registered at the General Post Office as a Newspaper.]

The Title-page and Index to Vol. III. is issued with this Number.

Per Annum, 6s. 6d., post free.

Monthly Parts, 6d.; post free, 7½d.

Ditto per Ann., 7s. 6d. post free.

